# *In vivo* efficacy of the boron-pleuromutilin AN11251 against *Wolbachia* of the rodent filarial nematode *Litomosoides sigmodontis*

**DOI:** 10.1371/journal.pntd.0007957

**Published:** 2020-01-27

**Authors:** Alexandra Ehrens, Christopher S. Lunde, Robert T. Jacobs, Dominique Struever, Marianne Koschel, Stefan J. Frohberger, Franziska Lenz, Martina Fendler, Joseph D. Turner, Stephen A. Ward, Mark J. Taylor, Yvonne R. Freund, Rianna Stefanakis, Eric Easom, Xianfeng Li, Jacob J. Plattner, Achim Hoerauf, Marc P. Hübner

**Affiliations:** 1 Institute for Medical Microbiology, Immunology and Parasitology, University Hospital Bonn, Bonn, Germany; 2 Anacor Pharmaceuticals, Palo Alto, California, United States of America; 3 Centre for Drugs and Diagnostics, Department of Tropical Disease Biology, Liverpool School of Tropical Medicine, Liverpool, United Kingdom; 4 German Center for Infection Research (DZIF), partner site Bonn-Cologne, Bonn, Germany; The University of Melbourne, AUSTRALIA

## Abstract

The elimination of filarial diseases such as onchocerciasis and lymphatic filariasis is hampered by the lack of a macrofilaricidal–adult worm killing–drug. In the present study, we tested the *in vivo* efficacy of AN11251, a boron-pleuromutilin that targets endosymbiotic *Wolbachia* bacteria from filarial nematodes and compared its efficacy to doxycycline and rifampicin. Doxycycline and rifampicin were previously shown to deplete *Wolbachia* endosymbionts leading to a permanent sterilization of the female adult filariae and adult worm death in human clinical studies. Twice-daily oral treatment of *Litomosoides sigmodontis*-infected mice with 200 mg/kg AN11251 for 10 days achieved a *Wolbachia* depletion > 99.9% in the adult worms, exceeding the *Wolbachia* reduction by 10-day treatments with bioequivalent human doses of doxycycline and a similar reduction as high-dose rifampicin (35 mg/kg). *Wolbachia* reductions of > 99% were also accomplished by 14 days of oral AN11251 at a lower twice-daily dose (50 mg/kg) or once-per-day 200 mg/kg AN11251 treatments. The combinations tested of AN11251 with doxycycline had no clear beneficial impact on *Wolbachia* depletion, achieving a > 97% *Wolbachia* reduction with 7 days of treatment. These results indicate that AN11251 is superior to doxycycline and comparable to high-dose rifampicin in the *L*. *sigmodontis* mouse model, allowing treatment regimens as short as 10–14 days. Therefore, AN11251 represents a promising pre-clinical candidate that was identified in the *L*. *sigmodontis* model, and could be further evaluated and developed as potential clinical candidate for human lymphatic filariasis and onchocerciasis.

## Author summary

Onchocerciasis and lymphatic filariasis are human filarial tropical diseases, which can cause blindness and severe dermatitis (onchocerciasis) or lymphedema and hydrocele (lymphatic filariasis). Current strategies to eliminate these diseases include the mass drug administration (MDA) of drugs that target the progeny of the filariae, the microfilariae, and temporarily inhibit filarial embryogenesis and, therefore, the transmission of the disease. However, MDA has several limitations that delay the goal of elimination including the lack of a drug with a short term regimen and a potent macrofilaricidal effect. As an alternative approach, the antibiotic doxycycline has been proven to be effective in depleting *Wolbachia* endosymbionts from adult filariae, which then leads to permanent sterilization and death of the adult worms. Due to contraindications for doxycycline and prolonged treatment regimen of at least 4 weeks, there is an urgent need for new anti-filarial drugs with an improved safety profile and shorter regimens. The current study demonstrates that the boron-pleuromutilin derivative AN11251 provides an excellent *in vivo* anti-*Wolbachia* depletion in the *Litomosoides sigmodontis* filarial mouse model that is superior to doxycycline and comparable to rifampicin, allowing for regimens as short as 10–14 days. Combination with doxycycline for 7 days had no significant beneficial effect on efficacy, achieving *Wolbachia* reductions of more than 97%. Therefore, AN11251 shows potent anti-*Wolbachia* activity in the *L*. *sigmodontis* mouse model and may also present an alternative pre-clinical candidate for filariasis treatment.

## Introduction

Onchocerciasis and lymphatic filariasis are neglected tropical diseases of humans [1, 2]. They represent debilitating diseases with severe dermatitis and vision loss (onchocerciasis) and lymphedema and hydrocele (lymphatic filariasis), leading to permanent disabilities, social stigmatization and income loss. These filarial diseases are considered a major public health and economic burden in endemic countries [3].

Current treatment strategies rely on mass drug administration (MDA) with ivermectin (IVM) for onchocerciasis and IVM in combination with albendazole (ALB) for lymphatic filariasis in sub-Saharan Africa [1, 2]. Recently, a newly described triple therapy with IVM, ALB and diethylcarbamazine (DEC) was evaluated for the elimination of lymphatic filariasis outside of Africa [4, 5] and is now recommended by the WHO for areas non-endemic for onchocerciasis [6]. However, the treatment strategies used for MDA have several limitations: (i) they predominantly target microfilariae (MF) and only temporarily inhibit the embryogenesis of female adult worms, which requires MDA treatment on an annual or bi-annual basis for the reproductive life span of the adult worms; (ii) modeling studies indicate that the reduction of MDA rounds will be less significant in areas of lower endemicity [5]; (iii) decreasing prevalence of onchocerciasis and lymphatic filariasis will reduce the cost effectiveness of community based MDA programs [7]; and (iv) risk of serious adverse events caused by DEC and IVM may hamper the implementation of the triple therapy in areas co-endemic for onchocerciasis and loiasis in Africa [3, 8, 9]. Therefore, new treatment strategies using macrofilaricidal compounds, i.e. compounds that kill adult filarial worms, are necessary for case management and for the “mopping up” of residual foci to achieve an elimination of both onchocerciasis and lymphatic filariasis.

As an alternative approach, endosymbiotic *Wolbachia* bacteria present in most human pathogenic filariae, including those causing onchocerciasis and lymphatic filariasis, can be targeted with antibiotics [10–12]. In human clinical trials doxycycline and rifampicin treatment have been shown to deplete *Wolbachia*, leading to the sterilization of female adult filariae of *Onchocerca volvulus* and *Wuchereria bancrofti* as well as to adult worm death [12–15]. Moreover, since *Loa loa* filariae do not possess endosymbiotic *Wolbachia*, drug-related serious adverse events in areas co-endemic for loiasis are unlikely to occur with *Wolbachia*-targeting drugs [9, 16]. However, for the treatment of lymphatic filariasis and onchocerciasis, doxycycline has to be given for at least 4 weeks to achieve a macrofilaricidal effect and permanent sterilization and doxycycline is contraindicated for pregnant or breastfeeding women as well as children of less than 8 years of age [3, 17, 18]. With regard to rifampicin, treatment with a low dose (10 mg/kg) over 4 weeks was shown to deplete *Wolbachia* in onchocerciasis patients, but the *Wolbachia* depletion was inferior to a doxycycline therapy for 6 weeks [19]. Conversely, high-doses of rifampicin were shown in preclinical models to deplete > 90% *Wolbachia* bacteria after 7 and 14 days of treatment in *Brugia malayi* and *Onchocerca ochengi*-infected animals, respectively [20]. The safety of such a high-dose has been supported by results of a human clinical trial for *Mycobacterium tuberculosis* [21]. Consequently, combinations of rifampicin with other drugs and/or rifampicin treatments at a higher dose may improve its efficacy and may allow shorter treatment regimens [15, 20].

Shortened treatment regimens may be also achieved by the discovery of novel *Wolbachia*-targeting drugs with an improved pharmacological profile. In the present study, we tested the efficacy of AN11251, an antibiotic of the pleuromutilin class, for its *in vivo*-efficacy against *Litomosoides sigmodontis–*a filarial nematode of rodents. Like doxycycline, pleuromutilins are ribosomal protein synthesis inhibitors, targeting predominantly Gram-positive bacteria [22]. The boron-pleuromutilin derivative AN11251 represents an anti-*Wolbachia* pre-clinical candidate that showed excellent pharmacokinetic properties and reduced *Wolbachia* loads in *B*. *malayi* and *L*. *sigmodontis* in rodents after oral administration [23]. Twice-daily treatment with 25 mg/kg AN11251 for 14 days achieved a *Wolbachia* reduction of 98.8% in *B*. *malayi* larvae and 16.6% *Wolbachia* reduction in *B*. *malayi* adults in SCID mice; Administration of 50 mg/kg AN11251 twice a day for 2 weeks resulted in a *Wolbachia* reduction of 99.7% in BALB/c mice infected with *L*. *sigmodontis* [23].

The aim of this study was to determine the minimal efficacious dose of AN11251 in the *L*. *sigmodontis* mouse model and to compare its efficacy with the bioequivalent human doses of rifampicin and doxycycline. The target candidate profile was an oral treatment regimen of 10 days or less, as this length of treatment was considered important to maximize the prospect of patient compliance in endemic areas. To compare the efficacy of the drugs, *L*. *sigmodontis* infected mice were treated by the oral route after the development of adult worms and the average *Wolbachia* depletion in adult worms as well as the frequency of female worms that achieved a 99% *Wolbachia* depletion was assessed 3–4 weeks after treatment start. Data from clinical trials with other anti-*Wolbachia* agents showed that such a *Wolbachia* reduction greater than 99% lead to a permanent sterilization of the female adult worms. Furthermore, the potential synergism of a combination therapy of doxycycline and AN11251 was evaluated to assess if it was possible to further reduce the treatment time required for *Wolbachia* depletion [24].

## Methods

### Ethics statement

All animal experiments were approved by the Landesamt für Natur, Umwelt und Verbraucherschutz, Köln, Germany, (AZ 84–02.04.2015.A507) and conducted in accordance with the European Union Directive 2010/63/UE. Animals were checked daily for food, water and welfare and the weight of each animal was determined once a week. Therefore, a score of A-C was assigned regarding the severity of any symptoms considering weight loss, behavioral change and appearance. Individual animals presented minor wounds due to territorial behavior (score A), but no drug-caused symptoms were observed in the treated animals. Necropsies were performed following an overdose of isoflurane (Abbot, Wiesbaden, Germany).

### Animals

Female BALB/c mice were obtained from Janvier (Le Genest-Saint-Isle, France) and were 7–8 weeks of age at the time of infection. Mice were housed in groups of five in individually ventilated cages (Tecniplast, West Chester, PA, USA) at the animal facility of the Institute for Medical Microbiology, Immunology and Parasitology, University Hospital Bonn. Food and water were provided *ad libitum* and animals were maintained on a 12 h light/dark cycle within a temperature range of 20–26°C and a relative humidity range of 30–70%.

### Test compounds

AN11251 was provided by Anacor Pharmaceuticals (Palo Alto, CA) and was > 98% pure [23]. The compound was formulated in 1% carboxymethyl cellulose (CMC) + 0.1% Tween80 or in 10% dimethyl sulfoxide (DMSO) (Sigma Aldrich, St. Louis, MO, USA) in phosphate-buffered saline (PBS)/1% CMC/0.1% Tween80 (see S1 Table). Aliquots of the formulated compound were stored at -20°C. Thawed aliquots were used within one day and kept in the refrigerator for this time. Doxycycline (Sigma Aldrich, St. Louis, MO, USA) was formulated in 10% DMSO in PBS or distilled water. Rifampicin (Sigma Aldrich, St. Louis, MO, USA) was prepared in polyethylene glycol 300 (PEG300)/propylene glycol/water at a volume ratio of 50/20/30 (S2 Table). Doxycycline and rifampicin were freshly prepared on each day. Control groups either received the vehicle 1% CMC/0.1% Tween or were left untreated.

### Study design and *L*. *sigmodontis* infection

The *L*. *sigmodontis* life cycle is maintained at the animal facility of the Institute for Medical Microbiology, Immunology and Parasitology, University Hospital Bonn. Female BALB/c mice were naturally infected via the tropical rat mite vector with *L*. *sigmodontis* as described elsewhere [25]. To ensure an equal infection, the same batch of mites were used for the infection of all animals within each experiment. At 35 days after infection (dpi), after the development into adult worms, mice were randomly assigned to treatment groups. Body weights of mice were determined daily during the treatment and the drugs were given in a volume of 10 ml/kg body weight. In a previous study, a 14-day twice-daily treatment of 50 mg/kg AN11251 was shown to deplete > 99% of *Wolbachia* in the *L*. *sigmodontis* mouse model [23]. In the current study, shorter treatment regimens of 7 and 10 days were tested in order to achieve the target candidate profile. The efficacy of different AN11251 doses and regimens were compared to rifampicin and doxycycline by analyzing the average *Wolbachia* depletion from female adult worms and the frequency of female worms that achieved a *Wolbachia* depletion of > 99.9%. In order to achieve shortened treatment regimens of AN11251, oral doses of 50, 100, 200, 300, and 400 mg/kg twice per day (*bis in die*, BID) or once per day (*quaque die*, QD) over a period of 7, 10 and 14 days (as indicated in S1 Table) were chosen. As positive controls, doxycycline was given at 40 mg/kg BID (2x 40 mg/kg per day), the predicted human bioequivalent dose [20], or 100 mg/kg QD (1x 100 mg/kg per day) for 7, 10 or 14 days. For rifampicin, QD doses of 10 and 35 mg/kg were administered for a duration of 7, 10 and 14 days. Oral vehicle administrations (1% carboxymethyl cellulose (CMC)/0.1% Tween80) or untreated animals served as negative controls (S1 Table, S2 Table). Since a combination of rifamycins and moxifloxacin was recently shown to allow shortened drug regimens [24], a combination therapy of AN11251 and doxycycline was tested. Therefore, for the combination therapy 40 mg/kg doxycycline and different AN11251 doses were used in a formulation of 10% DMSO in PBS/1% CMC/0.1% Tween80. 40 mg/kg doxycycline and 50 mg/kg AN11251 were both given BID for 14 days; 40 mg/kg doxycycline together with either 100 or 200 mg/kg AN11251 were administered BID for 7 days (S3 Table).

For pharmacokinetic (PK)/pharmacodynamic (PD) analysis, 50 μl of peripheral blood were taken from the *vena facialis* at 24 h and 48 h after the last gavage. Plasma was obtained by centrifugation at 1260 x g for 7 min at 4°C, and stored at -20°C; 14 days after the last gavage (i.e. 56, 64 dpi; see S1 Table, S2 Table and S3 Table), animals were euthanized by an overdose of isoflurane. Adult worms were isolated from the thoracic cavity by lavage, and the thoracic cavity as well as the peritoneum were screened for additional worms. Worms were counted, and female worms were analyzed for their motility and length; 10 female worms per mouse (n = 5 mice per treatment group) were individually frozen at -20°C for subsequent analysis of the *Wolbachia* FtsZ/*L*. *sigmodontis* actin ratio by duplex real-time PCR using Qiagen’s QuantiNova on a Rotorgene. The PCR was performed in triplicates as described previously [26]. Following primers were used: LsFtsZ FW (cgatgagattatggaacatataa), LsFtsZ RV (ttgcaattactggtgctgc), LsFtsZ TQP 5’6-FAM (cagggatgggtggtggtactggaa) 3’TAMRA, LsActin FW (atccaagctgtcctgtctct), LsActin RV (tgagaattgatttgagctaatg), LsActin TQP 5’HEX (actaccggtattgtgctcgatt) 3’TAMRA; 45 cycles were performed using an annealing temperature of 58°C. The standard for the duplex reaction was a combination of LsFtsZ and LsActin plasmids.

### Statistics

The primary efficacy parameter of the AN11251 treatment was based on the average *Wolbachia* reduction in female adult worms, which was measured by the FtsZ/actin ratio as well as the frequency of filariae that achieved a *Wolbachia* depletion > 99%. Therefore, the median *Wolbachia* FtsZ/*L*. *sigmodontis* actin ratio present at the time of necropsy in female adult worms of the treated groups was compared with that of the respective control group (vehicle control or untreated). To compare the percentage of the female adult worms that presented a *Wolbachia* reduction greater than 99% between the different regimens and treatments, the Chi-Quadrat-test was used to determine the Fisher’s exact test. As a secondary efficacy parameter the median adult worm burdens were compared between the treatment groups and the respective control groups. Data were analyzed for normal distribution using the Shapiro-Wilk normality test, and statistical analysis was performed using Kruskal-Wallis test with Dunn’s post-test for multiple groups.

## Results

### *Wolbachia* reduction by AN11251 at 200 mg/kg BID is superior to the standard bioequivalent human doses of rifampicin and doxycycline after 10 days of treatment

The aim of this study was to identify regimens that allow a shortened treatment with AN11251 for 7 or 10 days, which achieves a *Wolbachia* depletion of > 99% in mice harboring adult *L*. *sigmodontis* filariae and is therefore thought to lead to a permanent sterilization of the female adult worms. The efficacy of AN11251 treatment regimens on *Wolbachia* endosymbionts was compared to doxycycline and rifampicin treatments given for 7, 10 and 14 days starting from day 35 after infection as well as vehicle control treated/untreated animals.

**Fig 1:**
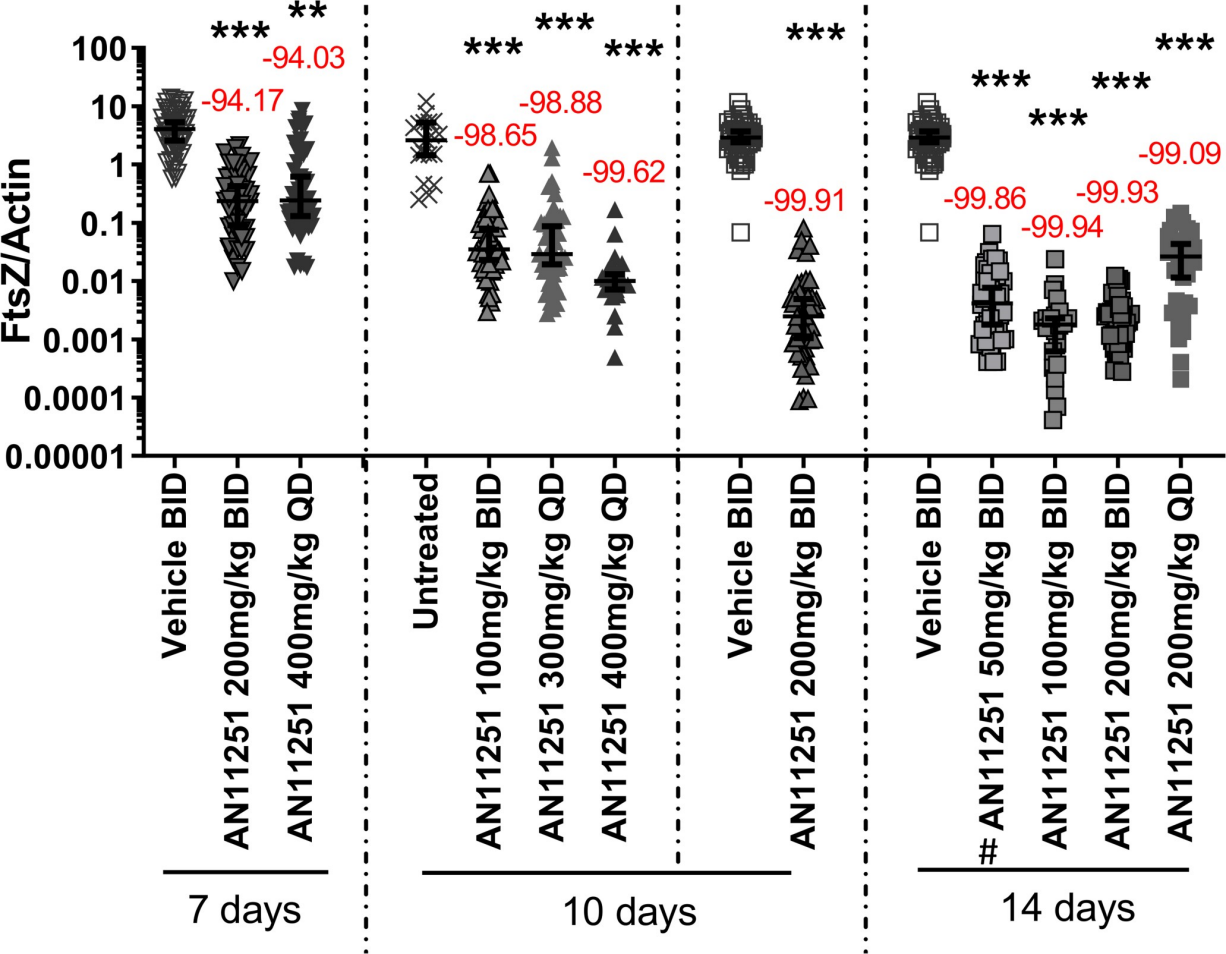
AN11251 reduces *Wolbachia* of *L*. *sigmodontis*. *Wolbachia* FtsZ/filarial actin ratio of female adult worms isolated from wild-type BALB/c mice that had been infected for 35 days with *Litomosoides sigmodontis* and treated with different concentrations of AN11251 (50, 100, 200, 300, and 400 mg/kg) for 7, 10 and 14 days or the vehicle control. The drug was given via the oral route as a twice-daily dosage (BID) or as a single dose per day (QD). Mice were sacrificed after 56 days of infection (dpi) (7-day treatment) or 64 dpi (10- and 14-day treatment). Results are shown as median with 95% Cl. N = 5 per group. Framed symbol: BID, symbol without frame: QD. **X**: untreated, black triangle upside down: 7-day treatment, black triangle upside: 10-day treatment, black square: 14-day treatment. #: already published data [23]. Statistical significance was analyzed by Kruskal-Wallis followed by Dunn‘s multiple comparison post-hoc test. **p<0.01; ***p<0.001.

The 7-day BID treatment with 200 mg/kg and 400 mg/kg AN11251 resulted in a *Wolbachia* FtsZ/actin reduction of 94.2% and 94.0%, respectively (Fig 1). Extended BID treatment for 10 days with 100 mg/kg and 200 mg/kg improved the *Wolbachia* FtsZ/actin reduction to 98.7% and 99.91%, respectively (Fig 1). Single daily doses of AN11251 for 10 days yielded a reduction of 98.9% and 99.6% when 300 mg/kg and 400 mg/kg AN11251 were given, respectively (Fig 1). These results indicate that 100 mg/kg AN11251 given twice a day for 10 days is sufficient to reduce *Wolbachia* within female worms by more than 98%. This reduction by 100 mg/kg AN11251 BID for 10 days is similar to a 10-day QD treatment with 300 mg/kg AN11251 resulting in a *Wolbachia* reduction of > 99% in 38.0% (19/50 worms) and 48.9% (22/45 worms) of female adult worms, respectively (Table 1; Fisher’s exact test p = 0.307). Prolonged treatment with AN11251 for 14 days BID further enhanced the *Wolbachia* FtsZ/actin reduction, achieving a reduction of 99.86%, 99.94%, and 99.93% with 50 mg/kg, 100 mg/kg, and 200 mg/kg AN11251, respectively (Fig 1). In the case of the 14-day BID treatment with 100 mg/kg and 200 mg/kg AN11251, *Wolbachia* reductions of > 99% were observed in all tested worms (Table 1). QD dosing of 200 mg/kg for 2 weeks resulted in a *Wolbachia* FtsZ/actin reduction of 99.1% (Fig 1). Thus, 14-day-treatment regimens with AN11251 at concentrations as low as 100 mg/kg BID as well as 10-day AN11251 treatment with 200 mg/kg BID were efficacious in clearing *Wolbachia* by > 99.9%, leading to a *Wolbachia* reduction of > 99% in all adult worms (at 100 mg/kg AN11251 BID for 14 days) or in 88% of adult worms (at 200 mg/kg AN11251 BID for 10 days) (Table 1; Fisher’s exact test p = 0.15).

**Table 1:** 14-day AN11251 treatment reduces the *Wolbachia* burden by > 99% in all adult worms analyzed. Shown are the number of female worms with less or more than 99% of *Wolbachia* reduction as well as the frequency of female worms, which presented a *Wolbachia* reduction greater than 99%. Female adult worms were isolated from wild-type BALB/c mice that had been infected for 35 days with *L*. *sigmodontis* and treated with different concentrations of AN11251 (50, 100, 200, 300, and 400 mg/kg), rifampicin (10 and 35 mg/kg) or doxycycline (40 and 100 mg/kg) for 7, 10 and 14 days or the vehicle control. The drug was given via the oral route as a twice-daily dosage (BID) or as a single dose per day (QD). AN11251: N = 5 per group, rifampicin and doxycycline: N = 4–5 animals per group (4 animals for 7 dpi doxycycline 40 mg/kg BID).

Treatment	Number of filaria with < 99% *Wolbachia* reduction	Number of filaria with > 99% *Wolbachia* reduction	Total number of worms analyzed	Percentage female filaria achieving a >99% *Wolbachia* reduction
7d BID AN11251 200 mg/kg	38	9	47	**19.2**
7d QD AN11251 400 mg/kg	43	5	48	**10.4**
10d BID AN11251 100 mg/kg	31	19	50	**38.0**
10d QD AN11251 300 mg/kg	23	22	45	**48.9**
10d QD AN11251 400 mg/kg	2	23	25	**92.0**
10d BID AN11251 200 mg/kg	5	37	42	**88.1**
14d BID AN11251 50 mg/kg	2	45	47	**95.7**
14d BID AN11251 100 mg/kg	0	24	24	**100.0**
14d BID AN11251 200 mg/kg	0	46	46	**100.0**
14d QD AN11251 200 mg/kg	18	18	36	**50.0**
7d QD rifampicin 10 mg/kg	36	4	40	**10.0**
7d QD rifampicin 35 mg/kg	41	2	43	**4.7**
10d QD rifampicin 10 mg/kg	16	21	37	**56.8**
10d QD rifampicin 35 mg/kg	6	34	40	**85.0**
14d QD rifampicin 10 mg/kg	1	47	48	**97.9**
14d QD rifampicin 35 mg/kg	0	31	31	**100.0**
7d BID doxycycline 40 mg/kg	43	1	44	**2.3**
10d BID doxycycline 40 mg/kg	40	8	48	**16.7**
10d BID doxycycline 100 mg/kg	45	3	48	**6.3**
14d BID doxycycline 40 mg/kg	2	36	38	**94.7**
14d QD doxycycline 100 mg/kg	14	35	49	**71.4**

BID = twice-daily dosage; QD = once per day dosage

**Fig 2:**
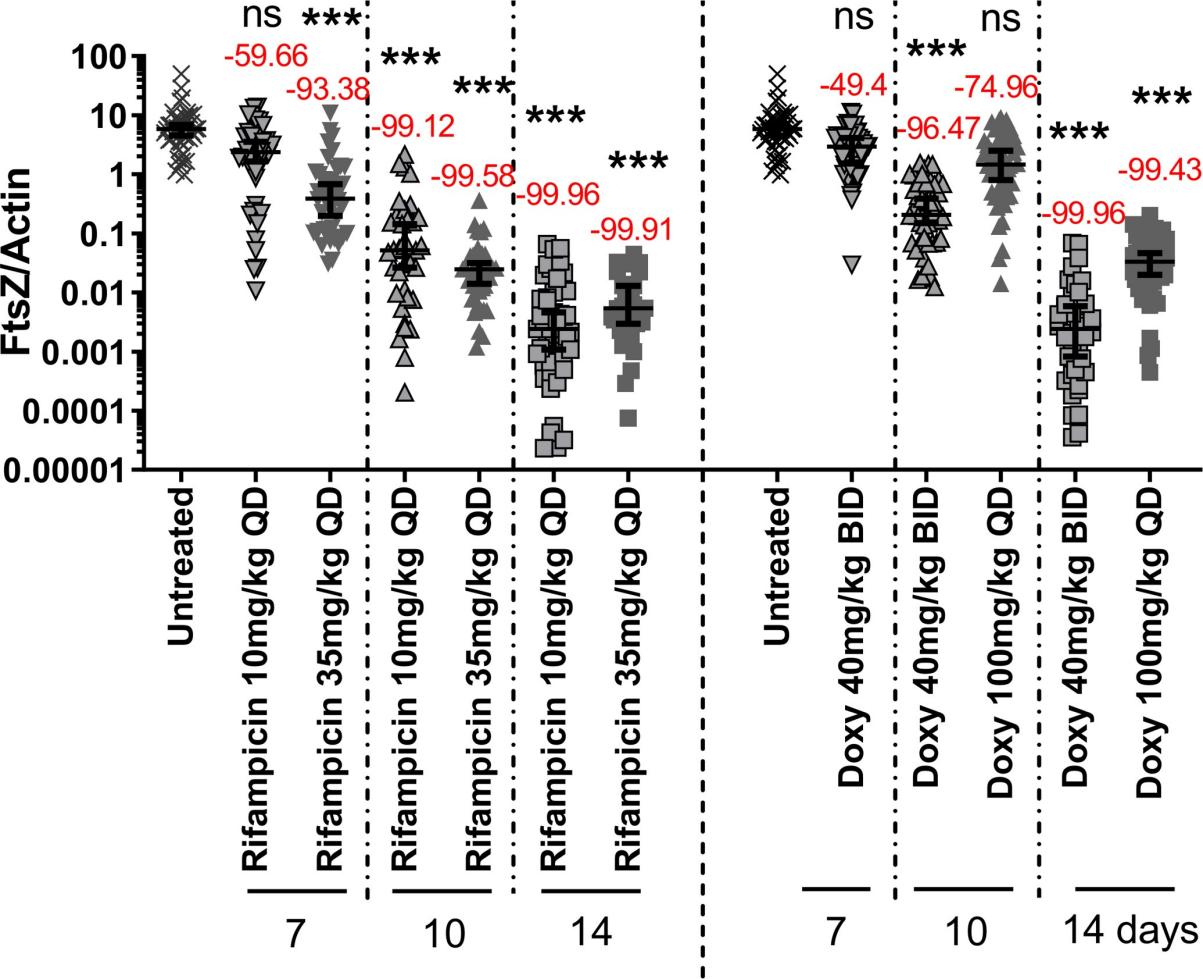
Rifampicin and doxycycline treatment reduces *Wolbachia* of *L*. *sigmodontis*. *Wolbachia* FtsZ/filarial actin ratio of female adult worms isolated from wild-type BALB/c mice that had been infected for 35 days with *Litomosoides sigmodontis* and treated with different rifampicin (10 and 35 mg/kg) or doxycycline (40 and 100 mg/kg) concentrations for 7, 10 and 14 days. Drugs were given via the oral route as a twice-daily dosage (BID) or as a single dose per day (QD). Mice were sacrificed after 64 days of infection (dpi); N = 4–5 animals per group (4 animals for 7 dpi doxycycline 40 mg/kg BID). Results are shown as median with 95% Cl. with frame N: untreated, black triangle upside down: 7-day treatment, black triangle up: 10-day treatment, black square: 14-day treatment. Statistical significance was analyzed by Kruskal-Wallis followed by Dunn‘s multiple comparison post-hoc test. ***p<0.001; ns = no statistical significance.

Rifampicin and doxycycline administered for 7 days at the standard human bioequivalent doses (rifampicin: 10 mg/kg QD; doxycycline: 40 mg/kg BID) and rifampicin at a high dose (35 mg/kg QD), resulted in an insufficient *Wolbachia* reduction (Fig 2); 40 mg/kg doxycycline given twice a day for 7 days reduced the *Wolbachia* FtsZ/actin ratio by 49.4% and by 96.5% after 10 days of treatment. After 14 days of BID treatment with 40 mg/kg doxycycline, the *Wolbachia* FtsZ/actin was reduced by 99.96% with 95% of adult worms showing a *Wolbachia* reduction > 99% (Table 1). QD treatments of 100 mg/kg doxycycline reduced the *Wolbachia* FtsZ/actin ratio by 75.0% and 99.4% after 10 and 14 days of treatment, respectively. This is consistent with the interpretation that a more potent efficacy is afforded with BID treatment at the shorter treatment regimens (Fig 2).

Rifampicin administered for 7 days at 10 mg/kg and 35 mg/kg QD resulted in a *Wolbachia* FtsZ/actin reduction of 59.7% and 93.4%, respectively. A *Wolbachia* FtsZ/actin reduction of > 2 logs was achieved by 10- and 14-day rifampicin administration (99.1% (10-day) and 99.96% (14-day) with the standard human bioequivalent dose of 10 mg/kg and 99.6% (10-day) and 99.91% (14-day) with the high dose of 35 mg/kg (Fig 2). These data indicate that the standard bioequivalent human and the high dose rifampicin are more efficacious at clearing *Wolbachia* bacteria from filarial nematodes than the bioequivalent human dose of doxycycline (10-day dosing: 10 mg/kg QD rifampicin 56.8% of worms with *Wolbachia* depletion > 99%; 35 mg/kg QD rifampicin 85% of worms with *Wolbachia* depletion > 99%; 40 mg/kg BID doxycycline 16.7% of worms with *Wolbachia* depletion > 99%; both comparisons Fisher’s exact test p<0.0001; Table 1). Table 2 summarizes the results from the *Wolbachia*-depletion with the different doxycycline and rifampicin treatment regimens. Fisher’s exact test indicates that 10 days of AN11251 at 200 mg/kg BID resulted in significantly (p = 0.002) more adult worms exhibiting a *Wolbachia* reduction of > 99% (37/42 worms) (Table 1) than 10 days of rifampicin treatment at 10 mg/kg QD (21/37 worms) and 10 days of BID 40 mg/kg doxycycline treatment (8/48 worms; p<0.0001) (Table 1). Administration of rifampicin at a high dose (35 mg/kg) for 10 days resulted in no statistical significant difference (Fisher’s exact test p = 0.753) in the number of filariae with a > 99% *Wolbachia* reduction (85%, 34/40 worms) compared to 10 days of AN11251 at 200 mg/kg BID treatment (88%, 37/42 worms) (Table 1).

**Table 2:** Summary of the *Wolbachia* reduction by AN11251, doxycycline and rifampicin. Reductions of the *Wolbachia* FtsZ/filarial actin ratio of female adult worms isolated from wild-type BALB/c mice that had been infected for 35 days with *L*. *sigmodontis* and treated with different concentrations of AN11251 (50, 100, 200, 300, and 400 mg/kg), doxycycline (40 and 100 mg/kg) or rifampicin (10 and 35 mg/kg) for 7, 10 and 14 days compared to the respective vehicle controls are shown.

	*Wolbachia* depletion in %		
	14-day treatment	10-day treatment	7-day treatment
**AN11251**			
50 mg/kg BID	99.9		
100 mg/kg BID	99.9	98.7	
200 mg/kg BID	99.9	99.9	94.2
200 mg/kg QD	99.1		
300 mg/kg QD		98.9	
400 mg/kg QD		99.6	94.0
**Doxycycline**			
40 mg/kg BID	100.0	96.5	39.7–49.4
100 mg/kg QD	99.4	75.0	
**Rifampicin**			
10 mg/kg QD	100.0	99.1	59.7
35 mg/kg QD	99.9	99.6	93.4
**Doxycycline+AN11251**			
40/50 mg/kg BID	99.9		
40/100 mg/kg BID			97.3
40/200 mg/kg BID			94.8

BID = twice-daily dosage; QD = once per day dosage

In conclusion, to achieve a *Wolbachia* reduction of > 99% in the majority of adult worms, AN11251 (200 mg/kg BID) and high-dose rifampicin (35 mg/kg QD) have to be given for 10 days, while doxycycline treatment must be administered for 14 days (Table 1).

### AN11251, doxycycline and rifampicin treatment regimens have no impact on adult worm burden in the *L*. *sigmodontis* mouse model

In addition to the *Wolbachia* FtsZ/actin reduction, the impact of different AN11251 concentrations on adult worm burden was assessed. These results show that none of the AN11251 treatment regimens tested resulted in a statistically significant adult worm reduction compared to the respective vehicle/untreated controls (S4 Table). Similarly, tested regimens of doxycycline and rifampicin did not lead to a significant adult worm reduction in the *L*. *sigmodontis* mouse model (S5 Table).

Thus, tested AN11251 as well as doxycycline and rifampicin regimens had no impact on the adult worm burden in the *L*. *sigmodontis* mouse model.

### Tested doxycycline and AN11251 combination regimens have no beneficial effect on *Wolbachia* FtsZ/actin reduction

**Fig 3:**
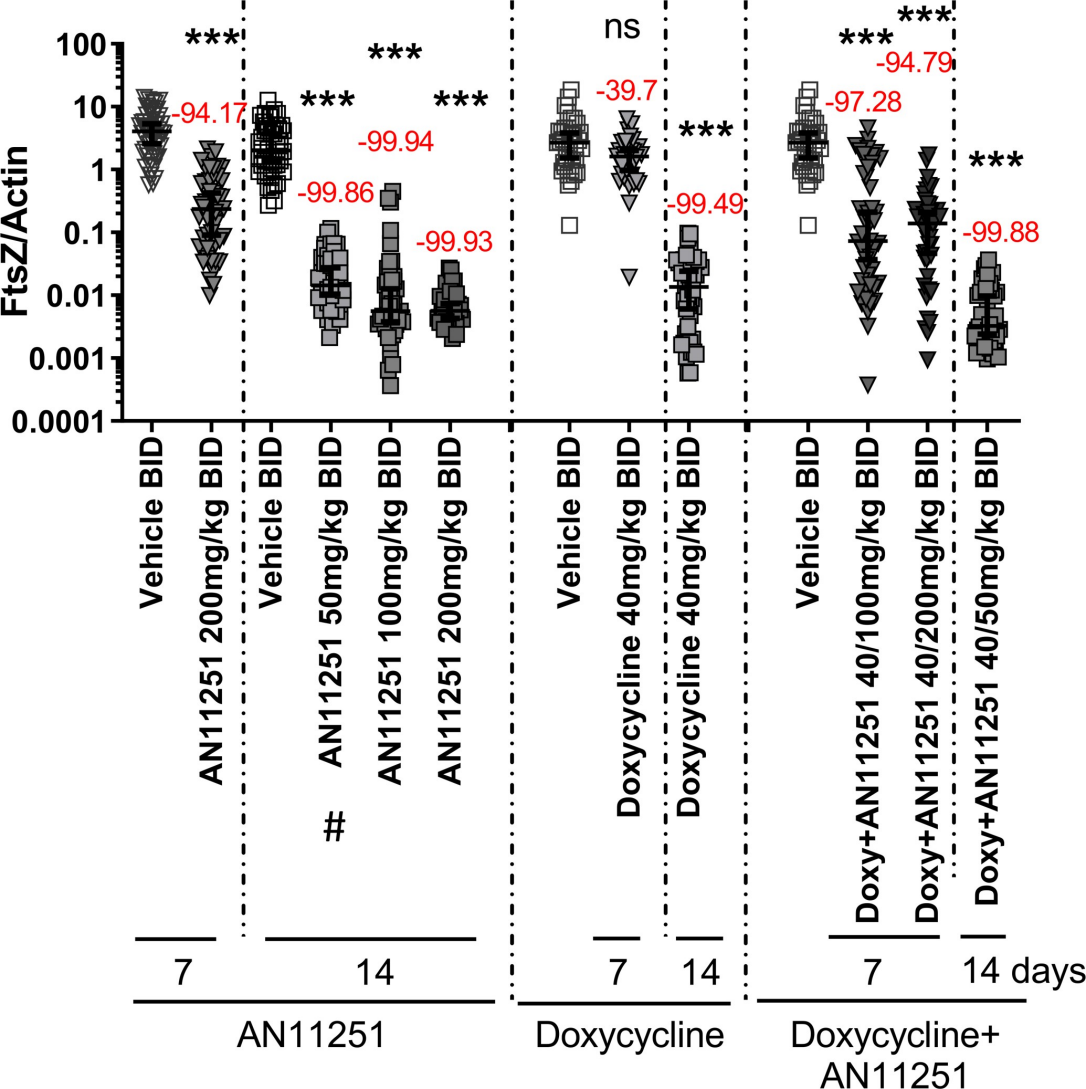
AN11251 and doxycycline combination therapy provides no improvement on *Wolbachia* reduction in comparison to the monotherapies. *Wolbachia* FtsZ/filarial actin ratio of female adult worms isolated from wild-type BALB/c mice that had been infected for 35 days with *L*. *sigmodontis* and treated with different AN11251 (50, 100, and 200 mg/kg) or doxycycline (40 mg/kg) (doxy) concentrations alone or in combination for 7 and 14 days. Drugs were given as a twice-daily oral dosage (BID). Shown is the FtsZ/actin ratio and the percent reduction. Results are shown as median with 95% Cl. N = 5. black triangle upside down: 7-day treatment, black square: 14-day treatment. #: already published data [23]. Statistical significance was analyzed by Kruskal-Wallis followed by Dunn‘s multiple comparison post-hoc test. ***p<0.001; ns = no statistical significance.

Results from the monotherapies showed that 10 days of AN11251 and 14 days of doxycycline treatment achieved a *Wolbachia* FtsZ/actin reduction of more than 99.9%. Therefore, we evaluated the potential of a combination therapy of doxycycline plus AN11251 to further reduce the treatment time required for *Wolbachia* depletion. Monotherapies for 7 days with AN11251 administered at 200 mg/kg BID resulted in a *Wolbachia* FtsZ/actin reduction of 94.2%, whereas the monotherapy with 40 mg/kg doxycycline BID reduced the *Wolbachia* FtsZ/actin ratio by 39.7% with a *Wolbachia* reduction of > 99% in 19.1% (9/47) and 2.3% (1/44) of female adult worms, respectively (Fig 3 and Table 3). A combination of 40 mg/kg doxycycline BID plus 100 mg/kg AN11251 BID for 7 days reduced *Wolbachia* loads by 97.3% compared to the vehicle controls, and achieved a *Wolbachia* reduction of > 99% in 31.9% (15/47) of female adult worms (Table 3). However, the combination of 40 mg/kg doxycycline BID plus 200 mg/kg AN11251 BID for 7 days achieved a similar *Wolbachia* reduction (94.8%) as the comparable monotherapy with AN11251 alone (94.2%), which led to a *Wolbachia* reduction of > 99% (Fisher’s exact test p = 0.797) in 23.3% (10/43) of female adult worms (Table 3). Furthermore, the 14-day treatment with 50 mg/kg AN11251 in combination with 40 mg/kg doxycycline did not lead to an improved *Wolbachia* reduction. This combination therapy resulted in a *Wolbachia* reduction of 99.88%, while the monotherapy of AN11251 reduced the *Wolbachia* by 99.86% and doxycycline treatment by 99.49% (Fig 3). The percentage of worms, which showed a *Wolbachia* reduction greater than 99% was also comparable between the combination therapy (95.2% of worms with *Wolbachia* reduction > 99%) and the monotherapies (AN11251: 95.7% and doxycycline: 94.7% of worms with *Wolbachia* reduction > 99%; Fisher’s exact test p = 1.0 for both comparisons; Fig 3 and Table 3). Similar to the monotherapies, the tested combination therapies had no impact on the adult worm burden (S6 Table). These data indicate that there is no essential beneficial impact on the depletion of *Wolbachia* endosymbionts with the tested AN11251 and doxycycline combination treatments.

**Table 3:** Tested AN11251 and doxycycline combination treatments provide no improvement on *Wolbachia* reduction in comparison to the monotherapies. Shown are number of female worms with less or more than 99% of *Wolbachia* reduction as well as the frequency of female worms, which presented a *Wolbachia* reduction greater than 99%. Female adult worms were isolated from wild-type BALB/c mice that had been infected for 35 days with *L*. *sigmodontis* and treated with different AN11251 (50, 100 and 200 mg/kg) or doxycycline (40 mg/kg) concentrations alone or in combination for 7 and 14 days. Drugs were given as a twice-daily oral dosage (BID). N = 5.

Treatment	Number of filaria with < 99% *Wolbachia* reduction	Number of filaria with > 99% *Wolbachia* reduction	Total number of worms analyzed	Percentage female filaria achieving a > 99% *Wolbachia* reduction
7d BID AN11251 200 mg/kg	38	9	47	**19.2**
14d BID AN11251 50 mg/kg	2	45	47	**95.7**
14d BID AN11251 100 mg/kg	0	24	24	**100.0**
14d BID AN11251 200 mg/kg	0	46	46	**100.0**
7d BID doxycycline 40 mg/kg	35	1	36	**2.8**
14d BID doxycycline 40 mg/kg	15	31	46	**67.4**
7d BID doxycycline/AN11251 40/100 mg/kg	32	15	47	**31.9**
7d BID doxycycline/AN11251 40/200 mg/kg	33	10	43	**23.3**
14d BID doxycycline/AN11251 40/50 mg/kg	2	40	42	**95.2**

BID = twice-daily dosage

## Discussion

Doxycycline, an antibiotic that targets endosymbiotic *Wolbachia*, has been proven to be an effective alternative treatment of onchocerciasis and lymphatic filariasis [11, 27, 28]. Doxycycline therapy depletes *Wolbachia* from adult filariae and MF, leading to permanent sterilization and adult worm death with treatment durations of 4–5 weeks in humans [10, 11, 14].

However, contraindications and the long treatment regimen impair a broader implementation of doxycycline as treatment for human filarial diseases, arguing for alternative anti-*Wolbachia* compounds with a higher potency allowing shorter treatment regimens. Rifampicin is a potential drug candidate, as it has been shown to reduce the *Wolbachia* burden in onchocerciasis patients after 4 weeks of treatment with the standard dose (10 mg/kg) regimen. Results achieved in animal models indicate that a higher dose of rifampicin of 30–35 mg/kg may enable a treatment regimen of 1–2 weeks for filarial diseases of humans [19, 20]. The safety profile of high rifampicin doses has been evaluated in human clinical trials against tuberculosis and appears to be safe up to a dose of 20 mg/kg and some additional studies also confirmed a safe profile with higher doses of 35 mg/kg [29–32].

In the current study, we compared the efficacy of AN11251, a boron-pleuromutilin, with rifampicin and doxycycline in its efficacy to deplete *Wolbachia* bacteria from *L*. *sigmodontis*. Pleuromutilins have been shown to be efficacious against Gram-positive bacteria and are frequently used to treat respiratory tract and skin infections [33], and the boron-pleuromutilin AN11251 has recently been described as an anti-*Wolbachia* drug candidate [23]. AN11251 shows good steady state exposure in plasma after oral administration with beneficial ADME (absorption, distribution, metabolism, and excretion) and pharmacokinetic properties [23]. At this point we do not have toxicology data on AN11251 beyond the observations that it is well tolerated at all the doses and durations tested. Furthermore, the range of doses explored with AN11251 are common for antibacterial efficacy studies with an investigational compound. The current study explored a comprehensive matrix of doses and dosing regimens to determine the minimal efficacious dose and treatment regimen of AN11251 required to deplete *Wolbachia* bacteria from *L*. *sigmodontis*. Administration of 200 mg/kg AN11251 BID for 10 days resulted in a *Wolbachia* reduction in the *L*. *sigmodontis* mouse model of > 99.9% with 88.1% of analyzed female worms showing a *Wolbachia* depletion of > 99% (Table 1), which is thought to lead to a permanent sterilization of female adult worms. Such a *Wolbachia* depletion by > 2 logs was observed in 16.7% of female adult worms after 10 days of 40 mg/kg BID doxycycline treatment, indicating that AN11251 is more potent than doxycycline in depleting *Wolbachia* endosymbionts (p<0.0001). Rifampicin, dosed at 10 and 35 mg/kg for 10 days, was superior to doxycycline, reaching a > 99% *Wolbachia* depletion after 10 days of treatment in 56.8% and 85% of female adult worms, respectively, highlighting also the improved efficacy of high-dose rifampicin. Thus, 10-day BID treatment with 200 mg/kg AN11251 resulted in a *Wolbachia* depletion that was comparable with high-dose rifampicin treatment for 10 days in the *L*. *sigmodontis* mouse model.

As combinations of rifamycins and moxifloxacin were recently shown to improve the clearance of *Wolbachia* in the *L*. *sigmodontis* mouse model and combinations of 2 weeks of doxycycline and rifampicin treatment resulted in a moderate macrofilaricidal effect in human *W*. *bancrofti* patients [15, 24], combinations of AN11251 and doxycycline were investigated in the present study. However, a combination of 100 mg/kg AN11251 with 40 mg/kg doxycycline did not significantly improve the *Wolbachia* depletion, achieving a *Wolbachia* depletion of 97% after 7 days of treatment. Thus, combinations of AN11251 with more potent antibiotics, such as rifampicin or moxifloxacin, should be explored for their potential to shorten treatment times and drug concentrations. Alternatively, as combinations with albendazole were recently shown to improve the efficacy of the *Wolbachia*-targeting drugs minocycline and rifampicin in *B*. *malayi*-infected jirds [34], combinations of AN11251 and albendazole may also improve efficacy, allowing shorter treatment times and lower doses.

Although AN11251, doxycycline and rifampicin were efficacious in depleting *Wolbachia* bacteria in the *L*. *sigmodontis*-mouse model, none of the treatment regimens tested revealed a macrofilaricidal efficacy. Lack of the macrofilaricidal efficacy in our study is likely due to the short time-frame between treatment and necropsy. The *L*. *sigmodontis* model is frequently used to test potential new drug candidates. However, the *L*. *sigmodontis* BALB/c mouse model has some limitations, since the majority of adult worms are cleared ~ 100 days after infection [35]. Thus, the treatment has to be initiated during the prepatent phase of filarial infection (before the onset of microfilaremia), which is normally not the case during human filarial infection. Furthermore, an impact of anti-*Wolbachia* drug candidates on adult worm burden and microfilariae counts is often not observed due to the slow macrofilaricidal efficacy of anti-*Wolbachia* compounds and the fact that only ~ 50% of mice develop microfilaremia [35], which is also seen in human lymphatic filariasis [36]. Nevertheless, the *L*. *sigmodontis* mouse model is very sensitive to short-course treatments and has repeatedly proven valuable for the screening of potential drug candidates against *Wolbachia* endosymbionts [24, 37–39] and provided the basis for the successful human clinical trials with doxycycline. Even though no macrofilaricidal effect was observed for the tested drugs, human clinical studies demonstrated that doxycycline provides a macrofilaricidal effect in human onchocerciasis patients only after 21–27 months following treatment [40].

In conclusion, this study provides further preclinical evidence that anti-*Wolbachia* drugs could be an effective therapy for human filarial diseases. Anti-*Wolbachia* drugs offer the opportunity to treat onchocerciasis in areas co-endemic for loiasis. Furthermore, anti-*Wolbachia* drugs have the potential for combination drug therapy, also with direct-acting macrofilaricides, to achieve greater efficacy and lessen the risk for drug resistance. AN11251 is such a potential anti-*Wolbachia* drug from a unique chemical class with improved efficacy compared with doxycycline and comparable efficacy to high-dose rifampicin in the *L*. *sigmodontis* model. AN11251 could be further evaluated and developed as potential backup clinical candidate for human lymphatic filariasis and onchocerciasis.

## Supporting information

S1 Table Experimental overview for AN11251 treatments. Drug and drug concentration, treatment duration and frequency, vehicle used, time point of analysis and number of animals per group are shown. Wild-type BALB/c mice have been infected for 35 days with *Litomosoides sigmodontis* and treated with different concentrations of AN11251 (50, 100, 200, 300 and 400 mg/kg) for 7, 10, 14 days. The drug was dissolved in 1% CMC/0.1% Tween80 or in 10% DMSO in PBS/1% CMC/0.1% Tween80 and given via the oral route as a twice-daily dosage (BID) or as a single dose per day (QD). Mice were sacrificed after 56 or 64 days of infection (dpi).
(DOCX)

S2 Table Experimental overview for doxycycline and rifampicin treatments. Drug and drug concentration, treatment duration and frequency, vehicle used, time point of analysis and number of animals per group are shown. Wild-type BALB/c mice have been infected for 35 days with *Litomosoides sigmodontis* and treated with different concentrations of doxycycline (40 or 100 mg/kg), rifampicin (10 or 35 mg/kg) or vehicle control/were left untreated for 7, 10 and 14 days. Rifampicin was dissolved in polyethylene glycol 300 (PEG300)/propylene glycol/water (50/20/30 volume ratio), while doxycycline was dissolved in aqua dest. or 10% DMSO in PBS. Drugs were given via the oral route as a twice-daily dosage (BID) or as a single dose per day (QD). Mice were sacrificed after 64 days of infection (dpi).
(DOCX)

S3 Table Experimental overview for AN11251 + doxycycline combination treatments. Drug and drug concentration, treatment duration and frequency, vehicle used, time point of analysis and number of animals per group are shown. Wild-type BALB/c mice have been infected for 35 days with *Litomosoides sigmodontis* and treated with doxycycline (40 mg/kg), AN11251 (50, 100 or 200 mg/kg) or vehicle control for 7 and/or 14 days. Combination therapy of AN11251 (50, 100, and 200 mg/kg) and doxycycline (40 mg/kg) was given for 7 and 14 days. Drugs were given via the oral route as a twice-daily dosage (BID). Mice were sacrificed after 56 or 64 days of infection (dpi).
(DOCX)

S4 Table AN11251 treatment has no impact on adult worm burden. Adult worm burden from wild-type BALB/c mice that have been infected for 35 days with *Litomosoides sigmodontis* and treated with different concentrations of AN11251 (50, 100, 200, 300 and 400 mg/kg) for 7, 10 and 14 days. Worm counts were determined at 56 days of infection (dpi) (7-day treatment) or 64 dpi (10 and 14-day treatment). Drugs were given via the oral route as a twice-daily dosage (BID) or as a single dose per day (QD). Shown is the median, Min-Max, mean, standard deviation (SD) and percent reduction of adult worm counts. Percent reduction was calculated from the median of the treatment group compared to the median of the control group. Analysis for statistical significance was done by Kruskal-Wallis followed by Dunn‘s multiple comparison post-hoc test.
(DOCX)

S5 Table Rifampicin and doxycycline treatment have no impact on adult worm burden. Adult worm burden from wild-type BALB/c mice that have been infected for 35 days with *Litomosoides sigmodontis* and treated with different rifampicin (10 and 35 mg/kg) or doxycycline (40 and 100 mg/kg) for 7, 10 and 14 days. Drugs were given via the oral route as a twice-daily dosage (BID) or as a single dose per day (QD). Worm counts were obtained at 64 days of infection (dpi). Shown is the median, Min-Max, mean, standard deviation (SD) and the percent reduction of the adult worm burden. Percent reduction was calculated from the median of the treatment group compared to the median of the control group. Analysis for statistical significance was done by Kruskal-Wallis followed by Dunn‘s multiple comparison post-hoc test.
(DOCX)

S6 Table AN11251 and doxycycline has no impact on adult worm load. Adult worm burden from wild-type BALB/c mice that have been infected for 35 days with *Litomosoides sigmodontis* and treated with different AN11251 (50, 100, and 200 mg/kg) or doxycycline (40 mg/kg) concentrations alone or in combination for 7 and 14 days. Drugs were given via the oral route as a twice-daily dosage (BID). Worm counts were determined at 56 or 64 days of infection (dpi). Shown is the median, Min-Max, mean, standard deviation (SD) and percent reduction of adult burden. Percent reduction was calculated from the median of the treatment group compared to the median of the control group. Analysis for statistical significance was done by Kruskal-Wallis followed by Dunn‘s multiple comparison post-hoc test.
(DOCX)
