# Supplementary material for: In vivo efficacy of the boron-pleuromutilin AN11251 against Wolbachia of the rodent filarial nematode Litomosoides sigmodontis
Source: PLoS Negl Trop Dis. 2020 Jan 27;14(1):e0007957. doi: 10.1371/journal.pntd.0007957 (PMC7004383; doi:10.1371/journal.pntd.0007957)
Supplement: S1 Table — Drug and drug concentration, treatment duration and frequency, vehicle used, time point of analysis and number of animals per group are shown. Wild-type BALB/c mice have been infected for 35 days with Litomosoides sigmodontis and treated with different concentrations of AN11251 (50, 100, 200, 300 and 400 mg/kg) for 7, 10, 14 days. The drug was dissolved in 1% CMC/0.1% Tween80 or in 10% DMSO in PBS/1% CMC/0.1% Tween80 and given via the oral route as a twice-daily dosage (BID) or as a single dose per day (QD). Mice were sacrificed after 56 or 64 days of infection (dpi). (DOCX) [file pntd.0007957.s001.docx]

| **Drug and Concentration** | **Dose per day** | **Duration (days)** | **Vehicle** | **Mice** | **End of Exp.** |
| --- | --- | --- | --- | --- | --- |
| Vehicle | BID | 7 | 1% CMC/0.1% Tween80 | 5 | 56 dpi |
| AN11251 200 mg/kg | BID | 7 | 1% CMC/0.1% Tween80 | 5 | 56 dpi |
| AN11251 400 mg/kg | QD | 7 | 1% CMC/0.1% Tween80 | 5 | 56 dpi |
| Untreated | - | - | - | 5 | 64 dpi |
| AN11251 100 mg/kg | BID | 10 | 10% DMSO in PBS/1% CMC/0.1% Tween80 | 6 | 64 dpi |
| AN11251 300 mg/kg | QD | 10 | 10% DMSO in PBS/1% CMC/0.1% Tween80 | 5 | 64 dpi |
| AN11251 400 mg/kg | QD | 10 | 10% DMSO in PBS/1% CMC/0.1% Tween80 | 6 | 64 dpi |
| Vehicle | BID | 14 | 1% CMC/0.1% Tween80 | 5 | 64 dpi |
| AN11251 200 mg/kg | BID | 10 | 1% CMC/0.1% Tween80 | 5 | 64 dpi |
| AN11251 50 mg/kg | BID | 14 | 1% CMC/0.1% Tween80 | 5 | 64 dpi |
| AN11251 100 mg/kg | BID | 14 | 1% CMC/0.1% Tween80 | 5 | 64 dpi |
| AN11251 200 mg/kg | BID | 14 | 1% CMC/0.1% Tween80 | 5 | 64 dpi |
| AN11251 200 mg/kg | QD | 14 | 1% CMC/0.1% Tween80 | 5 | 64 dpi |

BID = bi-daily dosage; QD = once per day dosage; CMC = Carboxymethyl cellulose; dpi = days post infection
