# Supplementary material for: In vivo efficacy of the boron-pleuromutilin AN11251 against Wolbachia of the rodent filarial nematode Litomosoides sigmodontis
Source: PLoS Negl Trop Dis. 2020 Jan 27;14(1):e0007957. doi: 10.1371/journal.pntd.0007957 (PMC7004383; doi:10.1371/journal.pntd.0007957)
Supplement: S2 Table — Drug and drug concentration, treatment duration and frequency, vehicle used, time point of analysis and number of animals per group are shown. Wild-type BALB/c mice have been infected for 35 days with Litomosoides sigmodontis and treated with different concentrations of doxycycline (40 or 100 mg/kg), rifampicin (10 or 35 mg/kg) or vehicle control/were left untreated for 7, 10 and 14 days. Rifampicin was dissolved in polyethylene glycol 300 (PEG300)/propylene glycol/water (50/20/30 volume ratio), while doxycycline was dissolved in aqua dest. or 10% DMSO in PBS. Drugs were given via the oral route as a twice-daily dosage (BID) or as a single dose per day (QD). Mice were sacrificed after 64 days of infection (dpi). (DOCX) [file pntd.0007957.s002.docx]

| **Drug and Concentration** | **Dose** | **Duration (days)** | **Vehicle** | **Mice** | **End of Exp.** |
| --- | --- | --- | --- | --- | --- |
| Untreated | - | - | - | 5 | 64 dpi |
| Rifampicin 10 mg/kg | QD | 7 | PEG300/Propylene glycol/water (50/20/30) | 5 | 64dpi |
| Rifampicin 35 mg/kg | QD | 7 | PEG300/Propylene glycol/water (50/20/30) | 5 | 64dpi |
| Rifampicin 10 mg/kg | QD | 10 | PEG300/Propylene glycol/water (50/20/30) | 5 | 64dpi |
| Rifampicin 35 mg/kg | QD | 10 | PEG300/Propylene glycol/water (50/20/30) | 5 | 64dpi |
| Rifampicin 10 mg/kg | QD | 14 | PEG300/Propylene glycol/water (50/20/30) | 5 | 64dpi |
| Rifampicin 35 mg/kg | QD | 14 | PEG300/Propylene glycol/water (50/20/30) | 5 | 64dpi |
| Doxy 40 mg/kg | BID | 7 | A. dest | 5 | 64 dpi |
| Doxy 40 mg/kg | BID | 10 | A. dest. | 5 | 64 dpi |
| Doxy 100 mg/kg | QD | 10 | A. dest. | 5 | 64 dpi |
| Doxy 40 mg/kg | BID | 14 | A. dest. | 5 | 64 dpi |
| Doxy 100 mg/kg | QD | 14 | A. dest. | 5 | 64 dpi |

Doxy = doxycycline; BID = bi-daily dosage; QD = once per day dosage; PEG = Polyethylene glycol; A. dest. = destilled water; CMC = Carboxymethyl cellulose; dpi = days post infection
