# Supplementary material for: In vivo efficacy of the boron-pleuromutilin AN11251 against Wolbachia of the rodent filarial nematode Litomosoides sigmodontis
Source: PLoS Negl Trop Dis. 2020 Jan 27;14(1):e0007957. doi: 10.1371/journal.pntd.0007957 (PMC7004383; doi:10.1371/journal.pntd.0007957)
Supplement: S3 Table — Drug and drug concentration, treatment duration and frequency, vehicle used, time point of analysis and number of animals per group are shown. Wild-type BALB/c mice have been infected for 35 days with Litomosoides sigmodontis and treated with doxycycline (40 mg/kg), AN11251 (50, 100 or 200 mg/kg) or vehicle control for 7 and/or 14 days. Combination therapy of AN11251 (50, 100, and 200 mg/kg) and doxycycline (40 mg/kg) was given for 7 and 14 days. Drugs were given via the oral route as a twice-daily dosage (BID). Mice were sacrificed after 56 or 64 days of infection (dpi). (DOCX) [file pntd.0007957.s003.docx]

| **Drug and Concentration** | **Dose per day** | **Duration (days)** | **Vehicle** | **Mice** | **End of Exp.** |
| --- | --- | --- | --- | --- | --- |
| Vehicle | BID | 7 | 1% CMC/0.1% Tween80 | 5 | 56 dpi |
| AN11251 200 mg/kg | BID | 7 | 1% CMC/0.1% Tween80 | 5 | 56 dpi |
| Vehicle | BID | 14 | 1% CMC/0.1% Tween80 | 5 | 64 dpi |
| AN11251 50 mg/kg | BID | 14 | 1% CMC/0.1% Tween80 | 5 | 64 dpi |
| AN11251 100 mg/kg | BID | 14 | 1% CMC/0.1% Tween80 | 5 | 64 dpi |
| AN11251 200 mg/kg | BID | 14 | 1% CMC/0.1% Tween80 | 5 | 64 dpi |
| Vehicle | BID | 14 | 1%CMC/0.1% Tween80 | 5 | 64 dpi |
| Doxy 40 mg/kg | BID | 7 | 10% DMSO in PBS | 5 | 64 dpi |
| Doxy 40 mg/kg | BID | 14 | 10% DMSO in PBS | 5 | 64 dpi |
| Doxy 40 mg/kg  AN11251 100 mg/kg | BID | 7 | 10% DMSO in PBS/1%CMC/0.1% Tween80 | 5 | 64 dpi |
| Doxy 40 mg/kg  AN11251 200 mg/kg | BID | 7 | 10% DMSO in PBS/1%CMC/0.1% Tween80 | 5 | 64 dpi |
| Doxy 40 mg/kg  AN11251 50 mg/kg | BID | 14 | 10% DMSO in PBS/1%CMC/0.1% Tween80 | 5 | 64 dpi |

Doxy = doxycycline; BID = bi-daily dosage; A. dest. = destilled water; CMC = Carboxymethyl cellulose; dpi = days post infection
