# Supplementary material for: In vivo efficacy of the boron-pleuromutilin AN11251 against Wolbachia of the rodent filarial nematode Litomosoides sigmodontis
Source: PLoS Negl Trop Dis. 2020 Jan 27;14(1):e0007957. doi: 10.1371/journal.pntd.0007957 (PMC7004383; doi:10.1371/journal.pntd.0007957)
Supplement: S5 Table — Adult worm burden from wild-type BALB/c mice that have been infected for 35 days with Litomosoides sigmodontis and treated with different rifampicin (10 and 35 mg/kg) or doxycycline (40 and 100 mg/kg) for 7, 10 and 14 days. Drugs were given via the oral route as a twice-daily dosage (BID) or as a single dose per day (QD). Worm counts were obtained at 64 days of infection (dpi). Shown is the median, Min-Max, mean, standard deviation (SD) and the percent reduction of the adult worm burden. Percent reduction was calculated from the median of the treatment group compared to the median of the control group. Analysis for statistical significance was done by Kruskal-Wallis followed by Dunn‘s multiple comparison post-hoc test. (DOCX) [file pntd.0007957.s005.docx]

|  |  |  |  |  | **Adult worm burden** | | | | | |
| --- | --- | --- | --- | --- | --- | --- | --- | --- | --- | --- |
| **Drug and Concentration** | **Dose** | **Duration (days)** | **End of Exp.** | **Mice** | **Median** | **Min - Max** | **Mean** | **SD** | **% reduction** | **Sign** |
| Untreated | - | - | 64 dpi | 5 | 31 | 22-52 | 33.0 | 11.3 | - | - |
| Rifampicin 10 mg/kg | QD | 7 | 64 dpi | 5 | 39 | 5-59 | 34.2 | 19.8 | -25.8 | ns |
| Rifampicin 35 mg/kg | QD | 7 | 64 dpi | 5 | 32 | 16-56 | 37.2 | 16.0 | -3.2 | ns |
| Rifampicin 10 mg/kg | QD | 10 | 64 dpi | 5 | 23 | 6-27 | 19.0 | 8.8 | 25.8 | ns |
| Rifampicin 35 mg/kg | QD | 10 | 64 dpi | 5 | 28 | 17-45 | 27.8 | 11.7 | 9.7 | ns |
| Rifampicin 10 mg/kg | QD | 14 | 64 dpi | 5 | 17 | 14-20 | 17.4 | 2.3 | 45.2 | ns |
| Rifampicin 35 mg/kg | QD | 14 | 64 dpi | 5 | 20 | 8-56 | 25.4 | 19.0 | 35.5 | ns |
| Untreated | - | - | 64 dpi | 5 | 31 | 22-52 | 33.0 | 11.3 | - | - |
| Doxy 40 mg/kg | BID | 7 | 64 dpi | 5 | 28 | 16-87 | 36.8 | 28.8 | 9.7 | ns |
| Doxy 40 mg/kg | BID | 10 | 64 dpi | 5 | 34 | 18-59 | 34.8 | 17.0 | -9.7 | ns |
| Doxy 100 mg/kg | QD | 10 | 64 dpi | 5 | 31 | 24-37 | 30.2 | 5.3 | 0.0 | ns |
| Doxy 40 mg/kg | BID | 14 | 64 dpi | 5 | 48 | 12-61 | 39.6 | 21.8 | -54.8 | ns |
| Doxy 100 mg/kg | QD | 14 | 64 dpi | 5 | 48 | 25-70 | 46.6 | 16.5 | -54.8 | ns |

Doxy = doxycycline; DPI = days post infection; SD = standard deviation; MF = microfilariae; Sign. = Statistical significance; ns = no statistical significance
