# Supplementary material for: In vivo efficacy of the boron-pleuromutilin AN11251 against Wolbachia of the rodent filarial nematode Litomosoides sigmodontis
Source: PLoS Negl Trop Dis. 2020 Jan 27;14(1):e0007957. doi: 10.1371/journal.pntd.0007957 (PMC7004383; doi:10.1371/journal.pntd.0007957)
Supplement: S6 Table — Adult worm burden from wild-type BALB/c mice that have been infected for 35 days with Litomosoides sigmodontis and treated with different AN11251 (50, 100, and 200 mg/kg) or doxycycline (40 mg/kg) concentrations alone or in combination for 7 and 14 days. Drugs were given via the oral route as a twice-daily dosage (BID). Worm counts were determined at 56 or 64 days of infection (dpi). Shown is the median, Min-Max, mean, standard deviation (SD) and percent reduction of adult burden. Percent reduction was calculated from the median of the treatment group compared to the median of the control group. Analysis for statistical significance was done by Kruskal-Wallis followed by Dunn‘s multiple comparison post-hoc test. (DOCX) [file pntd.0007957.s006.docx]

|  |  |  |  |  | **Adult worm burden** | | | | | |
| --- | --- | --- | --- | --- | --- | --- | --- | --- | --- | --- |
| **Drug and Concentration** | **Dose per day** | **Duration (days)** | **End of Exp.** | **Mice** | **Median** | **Min - Max** | **Mean** | **SD** | **% reduction** | **Sign** |
| Vehicle | BID | 7 | 56 dpi | 5 | 38 | 18-41 | 31.2 | 10.8 | - | - |
| AN11251 200 mg/kg | BID | 7 | 56 dpi | 5 | 27 | 17-31 | 25.0 | 5.5 | 29.0 | ns |
| Vehicle | BID | 14 | 64 dpi | 5 | 62 | 17-94 | 58.8 | 23.4 | - | - |
| AN11251 50 mg/kg | BID | 14 | 64 dpi | 5 | 26 | 13-74 | 35.0 | 23.9 | 58.1 | ns |
| AN11251 100 mg/kg | BID | 14 | 64 dpi | 5 | 23 | 2-44 | 22.8 | 18.9 | 62.9 | ns |
| AN11251 200 mg/kg | BID | 14 | 64 dpi | 5 | 24 | 18-100 | 42.2 | 34.9 | 61.3 | ns |
| Vehicle | BID | 14 | 64 dpi | 5 | 21 | 11-59 | 30.0 | 21.4 | - | - |
| Doxy 40 mg/kg | BID | 7 | 64 dpi | 4 | 28 | 19-33 | 27.0 | 5.9 | -33.3 | ns |
| Doxy 40 mg/kg | BID | 14 | 64 dpi | 5 | 27 | 10-34 | 24.4 | 10.3 | -28.6 | ns |
| Vehicle | BID | 14 | 64 dpi | 5 | 21 | 11-59 | 30.0 | 21.4 | - | - |
| Doxy + AN11251 40/100 mg/kg | BID | 7 | 64 dpi | 5 | 43 | 22-66 | 42.4 | 18.0 | -104.8 | ns |
| Doxy + AN11251 40/200 mg/kg | BID | 7 | 64 dpi | 5 | 50 | 19-57 | 42.2 | 15.5 | -138.1 | ns |
| Doxy + AN11251 40/50 mg/kg | BID | 14 | 64 dpi | 5 | 38 | 5-74 | 38.4 | 24.5 | -81.0 | ns |

Doxy = doxycycline; DPI = days post infection; SD = standard deviation;

Sign. = Statistical significance; ns = no statistical significance
